# Optimizing Recovery in Elderly Patients: Anabolic Benefits of Glucose Supplementation during the Rehydration Period

**DOI:** 10.3390/nu16111607

**Published:** 2024-05-24

**Authors:** Ondrej Sobotka, Vojtech Mezera, Vladimir Blaha, Pavel Skorepa, Joao Fortunato, Lubos Sobotka

**Affiliations:** 1 3rd Department of Internal Medicine-Metabolism and Gerontology, University Hospital Hradec Kralove, 500 05 Hradec Kralove, Czech Republic; sobotkao@lfhk.cuni.cz (O.S.); pustik@lfhk.cuni.cz (L.S.); 2 Oroboros Instruments, 6020 Innsbruck, Austria; 3 Faculty of Medicine in Hradec Kralove, Charles University, 500 03 Hradec Kralove, Czech Republic; 4 Geriatric Center, Pardubice Hospital, 532 03 Pardubice, Czech Republic; 5 Department of Military Internal Medicine and Military Hygiene, Military Faculty of Medicine, University of Defence, 500 01 Hradec Kralove, Czech Republic

**Keywords:** glucose, dehydration, malnutrition, refeeding syndrome

## Abstract

Background: Since many acutely admitted older adults display signs of dehydration, treatment using balanced crystalloids is an important part of medical care. Additionally, many of these patients suffer from chronic malnutrition. We speculated that the early addition of glucose might ameliorate the hospital-related drop of caloric intake and modify their catabolic status. Methods: We included patients 78 years and older, admitted acutely for non-traumatic illnesses. The patients were randomized into either receiving balanced crystalloid (PlasmaLyte; group P) or balanced crystalloid enriched with 100 g of glucose per liter (group G). The information about fluid balance and levels of minerals were collected longitudinally. Results: In the G group, a significantly higher proportion of patients developed signs of refeeding syndrome, i.e., drops in phosphates, potassium and/or magnesium when compared to group P (83.3 vs. 16.7%, *p* < 0.01). The drop in phosphate levels was the most pronounced. The urinalysis showed no differences in the levels of these minerals in the urine, suggesting their uptake into the cells. There were no differences in the in-hospital mortality or in the 1-year mortality. Conclusion: The short-term administration of balanced crystalloids with glucose induced an anabolic shift of electrolytes in acutely admitted older adults.

## 1. Introduction

Recent studies indicate that a significant proportion of older adults acutely admitted to hospitals exhibit signs of dehydration, with prevalence rates reported up to 38% [1,2] which further exacerbate urological, gastrointestinal, circulatory and neurological disorders [3]. Consequently, the administration of balanced crystalloids has become a cornerstone of standard medical care for these patients [4,5,6,7]. 

Additionally, chronic malnutrition, prevalent among this demographic group of patients [8,9,10], often precipitates catabolic states, leading to protein degradation and consequent muscle wasting [11]. Sarcopenia, in the context of chronic malnutrition, results from a complex interplay of decreased food intake, altered hormone levels, and inflammation [12]. Subsequently, the body’s energy reserves are depleted, leading to an increased breakdown of body proteins to supply amino acids for gluconeogenesis to synthesize glucose from non-carbohydrate sources [13]. This process is regulated by catabolic hormones, further accelerating muscle protein degradation. Concurrently, the reduction in anabolic signals, primarily from insulin and growth hormone, diminishes protein synthesis [12,13]. This condition is further compounded by inflammation [14] and diminished dietary intake during hospitalization [9,15,16], contributing to a loss in muscle mass—an outcome associated with decreased autonomy, even loss of self-sufficiency [17]. 

Glucose, a fundamental substrate, plays a pivotal role in anabolic processes, the immune response [18,19] and the growth and differentiation of cells [20]. Specifically, glucose contributes to the reductive synthesis of amino acids and proteins via the pentose phosphate pathway, enhancing nonoxidative glucose metabolism [21,22].

Glucose supplementation activates key metabolic pathways that counteract the catabolic state induced by malnutrition and illness. Primarily, glucose stimulates insulin release, a hormone with potent anabolic effects [23]. Insulin promotes glucose uptake by muscle and other tissues, stimulating glycogen synthesis and inhibiting proteolysis. Additionally, glucose is metabolized through the pentose phosphate pathway, producing nicotinamide adenine dinucleotide phosphate (NADPH), a critical cofactor in reductive biosynthesis reactions, including the synthesis of fatty acids and nucleotides [21,24,25]. This pathway also generates ribose-5-phosphate, necessary for nucleotide synthesis, supporting DNA repair and replication [22,24]. Furthermore, glucose-derived pyruvate can enter the Krebs cycle, leading to the production of adenosine triphosphate (ATP) and precursors of amino acid synthesis, facilitating the shift towards protein synthesis and muscle growth [23]. These metabolic effects of glucose not only support the maintenance of muscle mass but also enhance the immune response and cellular repair processes, crucial for recovery in hospitalized patients [18,19]. 

The hypothesis that glucose supplementation could initiate anabolic processes and insulin secretion is supported by evidence showing that carbohydrate intake in individuals who have experienced starvation due to illness decreases plasma phosphate levels but also stimulates whole-body protein synthesis [26]. 

The initiation of nutritional support in chronically malnourished patients is often connected with the risk of refeeding syndrome [1,27], which is characterized by the shift of phosphates and other extracellular electrolytes into cells, with a subsequent drop in plasma levels that potentially leads to cardiac and neurological complications, as well as increased mortality [28,29]. However, refeeding syndrome is an indicator of anabolism, which is a positive consequence of nutritional support. However, if this condition is undetected, it leads to the complications mentioned above. Therefore, frequent laboratory monitoring [4,29], particularly within the first 72 h of the treatment, and early supplementation are mandatory in formerly depleted patients [30,31]. 

The aim of the present study was to analyze serum ion concentration changes during the rehydration of elderly patients admitted to a non-intensive care internal medical department due to acute non-traumatic illnesses. The patients were monitored during the first 7 days of their hospital stays, with a special emphasis on the first 72 h after admission. We tried to answer the question whether glucose administered together with rehydration therapy can induce cellular anabolism, which is manifested by a change in plasma minerals, which is typical for refeeding syndrome.

## 2. Materials and Methods

Our study was performed at an acute geriatric ward of the 3rd Department of Internal Medicine-Metabolism and Gerontology, University Hospital Hradec Kralove, which is a tertiary hospital. Patients who were acutely admitted for non-traumatic illnesses and fulfilled the inclusion criteria were offered a chance to participate in the study. The inclusion criteria were as follows: aged 78 years and older, assumption of hospital stay longer than 3 days, clinical/physical signs of insufficient hydration at admission, and willingness to participate with a signature representing informed consent. The exclusion criteria were as follows: newly diagnosed heart failure, decompensated diabetes mellitus with either ketoacidosis or glycaemia 20 mmol L^−1^ and higher, terminal illness with indication for supportive treatment only, and the patient not providing informed consent. The study protocol was approved by the Ethics Committee of the University Hospital in Hradec Králové under number 201805 S12P, on 3 May 2018.

At the admission, each patient has had his/her hydration status assessed by both clinical and laboratory methods. The clinical/physical signs were a feeling of thirst, dry mucous membranes, dark urine, low skin turgor, tachycardia with a heart rate over 100 beats per minute, hypotension with systolic blood pressure below 100 mmHg and sunken eye bulbs [6]. 

In cases of insufficient oral fluid intake, intravenous hydration was initiated according to group assignment. The patients were randomly divided (randomization using numbered and sealed envelopes) into two groups. The two arms received either balanced crystalloid solution (PlasmaLyte; group P) or the same balanced crystalloid solution enriched with 100 g of glucose per liter (group G). The PlasmaLyte solutions were obtained from Baxter Czech, Prague, Czech Republic, and the addition of glucose and solutes was performed under sterile conditions at the hospital pharmacy. The composition of the solutions is provided in Table 1.

Together with the crystalloids, oral fluids were offered (plain or sweetened tea, mineral water, juices, etc.). The amount and composition of administered fluids was adjusted by the attending physician, based on clinical status and lab test results. When plasma levels of electrolytes were low or decreased during treatment, these were added into the rehydration solutions with the aim of preventing depletion. When parenteral nutrition was indicated, a parenteral nutrition was administered (Nutriflex^®^ Peri for patients with peripheral catheters or Nutriflex^®^ Plus for patients with central venous catheters—both produced by B. Braun, Melsungen, Germany).

The information about fluid balance and levels of minerals were collected longitudinally. Fluid intake was calculated together with the number of individual constituents administered. Patients’ venous blood was collected at admission, then on the second, third, fourth, fifth and seventh days of their hospital stay. The following parameters were analyzed: serum levels of sodium, potassium, chloride, phosphates and magnesium; nitrogenous catabolites; urea and creatinine; liver injury; synthetic function markers alanine aminotransferase (ALT), aspartate aminotransferase (AST) and alkaline phosphatase (ALP); bilirubin; albumin; glycaemia; and markers of inflammation C-reactive protein (CRP) and leukocyte count. 

Urine output was measured reliably only in patients with introduced urinary catheter (n = 8 in the P group, n = 5 in the G group). The decision about inserting a urinary catheter relied solely on the physician in charge of the patient, mostly in patients with markedly decreased mobility. Our choice to not force the physician to introduce urinary catheters was based on the fact that the presence of catheters increases the risk of the patient developing delirium and the related risk of self-harm. 

The presence of refeeding syndrome was diagnosed according to Friedli et al. [11,32], as follows: 1. a decrease in phosphate levels by 30% from the baseline, 2. a decrease in phosphate levels below 0.6 mmol L^−1^, and 3. a decrease in two electrolytes below the normal range: magnesium below 0.75 mmol L^−1^, phosphates below 0.8 mmol L^−1^ and potassium below 3.5 mmol L^−1^. 

We processed the data using Sheets (Google LLC, Mountain View, CA, USA) and Excel 2010 (Microsoft Corporation, Seattle, WA, USA). The Chi-squares tests and multiple linear regressions were performed using Prism 10.2.2 (GraphPad Software, La Jolla, CA, USA). The data are given as percentages of the respective groups, as well as absolute numbers, in the case of frequency data. The continuous variables are shown as median (interquartile—IQ—range in the parentheses). To analyze the changes in individual minerals and not miss data from patients with blood withdrawals on different days, we calculated a 3- and 7-day slope by means of simple linear regression (Supplementary Figure S1) and used the slopes instead of individual values. The excretion of individual electrolytes was analyzed using a two-way analysis of variance (ANOVA) using a grouped arrangement. *p* < 0.05 was set as a threshold of significance for all analyses.

## 3. Results

### 3.1. Cohort Characteristics

We included a total of 34 patients. These patients were not able to take in sufficient amounts of fluids orally and required intravenous fluid therapy. Of those, 18 received balanced crystalloids (group P) and 16 received crystalloids enriched with 100 g L^−1^ glucose (group G). The cohort characteristics are provided in Table 2. 

### 3.2. The Occurrence of Refeeding Syndrome

**Figure 1:**
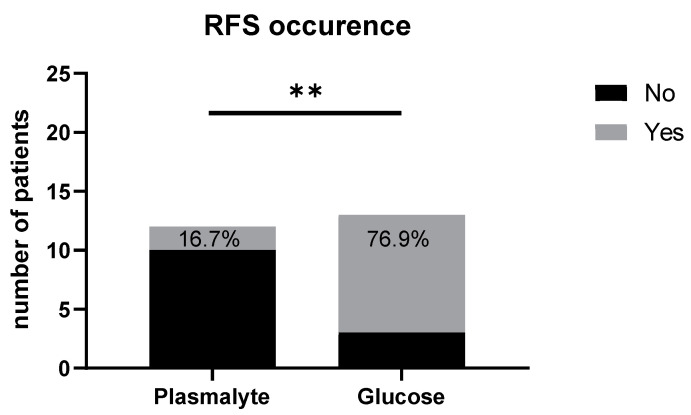
Comparison of the occurrence of refeeding syndrome (RFS) in the patients receiving balanced crystalloid (PlasmaLyte) and in the patients receiving balanced crystalloid enriched with glucose (glucose). The refeeding syndrome occurred in 16.7% of patients in the PlasmaLyte group and in 76.9% in the glucose group (** *p* < 0.01).

In the G group, a significantly higher proportion of patients developed signs of refeeding syndrome, i.e., a drop in phosphates, potassium and/or magnesium [32] when compared to group P (76.9% vs. 16.7%, *p* < 0.01, Figure 1). 

This result was also confirmed by a multiple logistic regression (*p* < 0.05) using the following variables: group (G or P), age, baseline potassium levels, baseline magnesium levels, baseline phosphate levels, baseline urea and creatinine levels and baseline CRP levels.

### 3.3. Trends of Individual Electrolytes in the Serum

**Figure 2:**
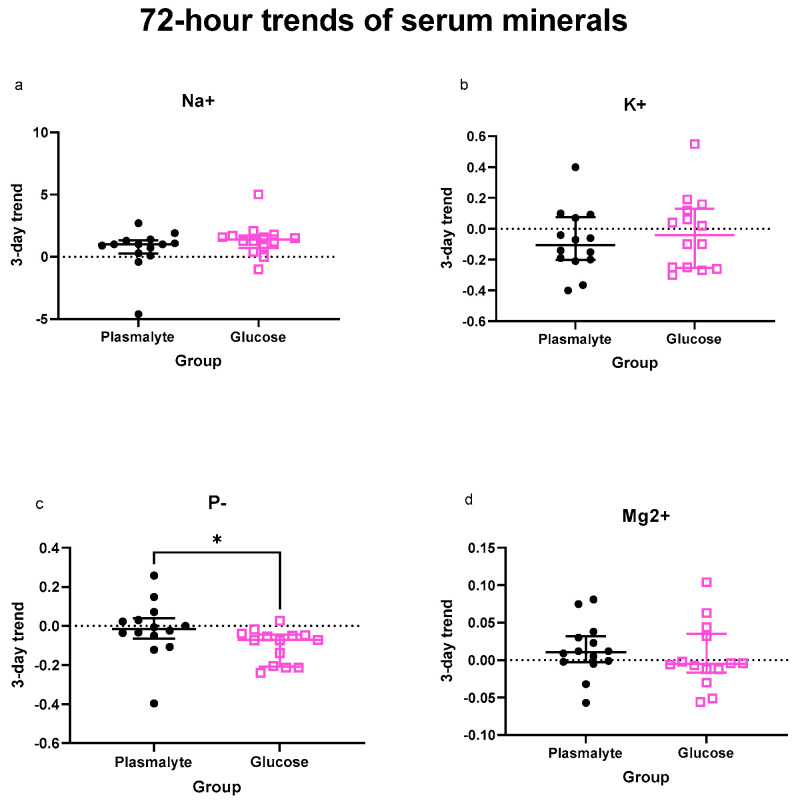
Comparison of 72 h slopes of (**a**) natrium, (**b**) potassium, (**c**) phosphates and (**d**) magnesium levels. Scatter plots show median and interquartile range. * *p* < 0.05.

**Figure 3:**
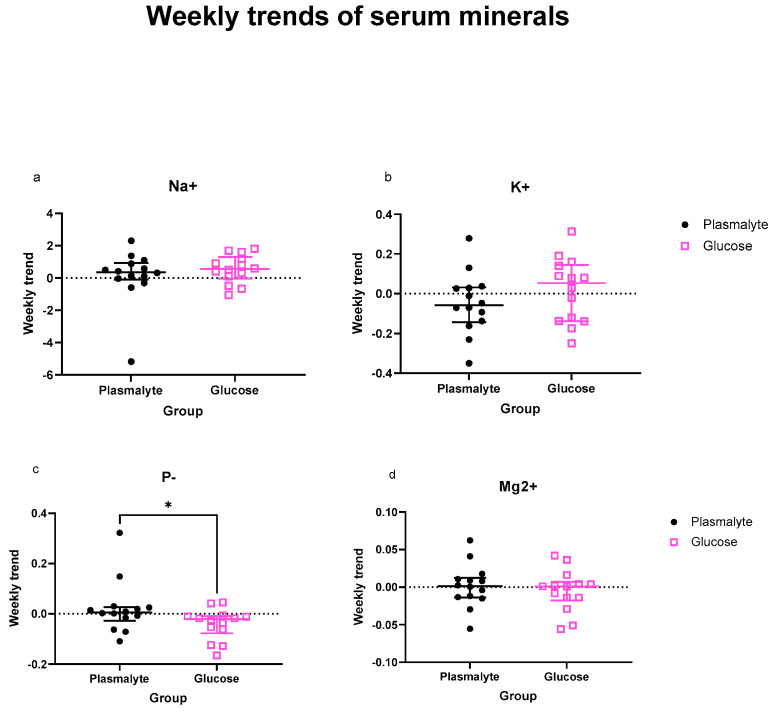
Comparison of one-week slopes of (**a**) natrium, (**b**) potassium, (**c**) phosphates and (**d**) magnesium levels. Scatter plots show median and interquartile range. * *p* < 0.05.

When analyzing the levels of individual electrolytes, the drop in phosphate levels in the G group was the most pronounced and was the only variable reaching significant difference from the P group over both 72 h and one week (Figure 2c and Figure 3c).

When analyzing the levels of electrolytes as trends in the entire groups, the only significant decrease was observed in phosphate levels during the first 72 h (Supplementary Figure S2d) but not during the first week (Supplementary Figure S3).

The detailed comparison of electrolyte substitution is provided in Supplementary Figure S4. Notably, the patients in the G group more frequently required substitution of potassium, phosphates (both *p* < 0.001) and magnesium (*p* < 0.01). 

### 3.4. Electrolytes in the Urine

**Figure 4:**
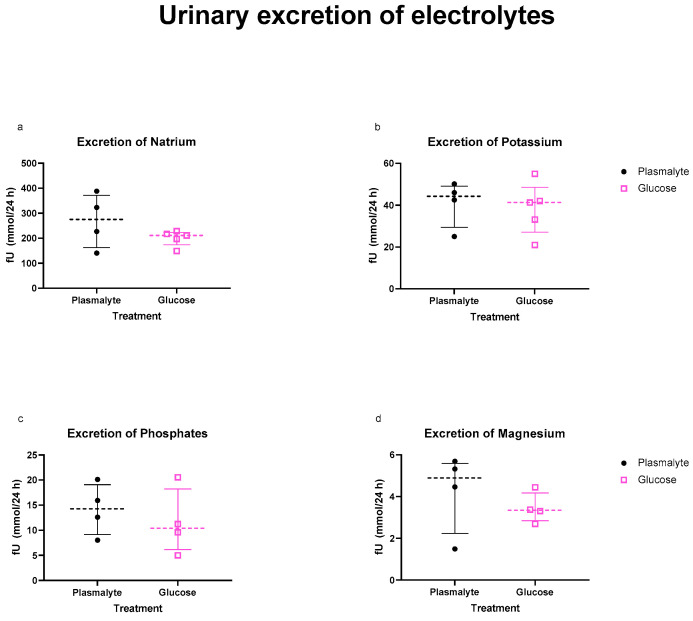
Comparison of urinary excretion of (**a**) natrium, (**b**) potassium, (**c**) phosphates and (**d**) magnesium. Analyzed only in the patients with a urinary catheter. Scatter plots show median and interquartile range.

The urinalysis showed no differences in the levels of any of the minerals of interest (Figure 4). 

### 3.5. Markers of Inflammation

There was a nonsignificant trend of higher CRP levels at baseline in the P group compared to the G group (Table 2). The weekly slopes of CRP levels and leukocyte counts did not differ among the groups. Patients in both groups displayed a mostly gradual decline in both markers (Supplementary Figure S5).

### 3.6. Further Patients’ Follow Up

**Figure 5:**
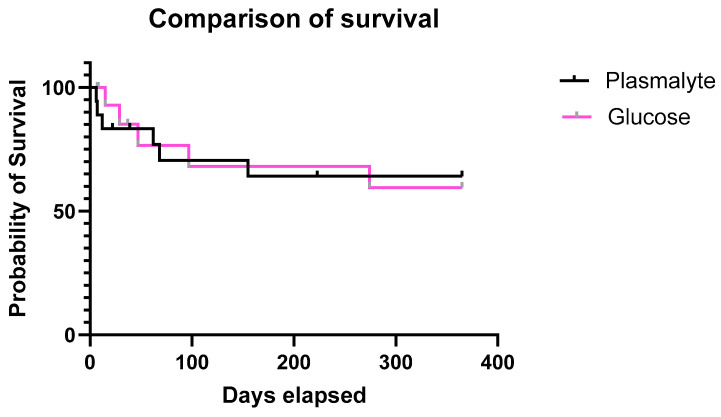
Analysis of one-year survival of the patients receiving balanced crystalloid (PlasmaLyte) and in the patients receiving balanced crystalloid enriched with glucose (Glucose).

There were no differences in mortality during a 1-year follow-up (*p* = 0.812, Figure 5).

## 4. Discussion

Intravenous infusion therapy is a critical component of care for acutely hospitalized patients, especially geriatric individuals who often present with significant dehydration. Guidelines on the use of crystalloid solutions, with or without glucose, are shaped by specific clinical contexts. Although hyperglycemia is commonly interpreted as a negative prognostic indicator, its association in acute settings may be more linked to reduced insulin sensitivity from acute pathologies rather than glucose administration itself.

Our study found that early glucose supplementation did not significantly affect mortality rates or alter one-year mortality outcomes, though it did reduce hospital stay duration from 11.5 to 9 days. The analysis of inflammation markers such as CRP and leukocyte counts showed no significant differences between groups, supporting previous findings [19] that challenge the assumed risks of glucose in rehydration and parenteral nutrition.

We observed that glucose-enriched crystalloids significantly lowered serum phosphate levels and slightly reduced potassium and magnesium levels, which could be an indicator of anabolic situation, misinterpreted as refeeding syndrome (RFS). 

The development of RFS, characterized by a decline in levels of potassium, magnesium and phosphate, occurred in the majority of recipients [11,32]. When utilizing established malnutrition screening tools, we found that over three-quarters of malnourished geriatric hospitalized patients exhibited a significant risk of RFS [8]. A decrease in phosphate levels was noted in 15% to 25% of these patients [30]. Despite a generally low risk associated with enteral feeding [33], RFS was also observed in patients receiving this form of nutrition [31].

However, RFS also developed in 16.7% of our patients receiving non-glucose solutions, suggesting that RFS is not exclusively linked to glucose supplementation.

An additional 100 g of glucose provides 390 kCal (1630 kJ) [34], translating to 21.7 kJ/kg for an average 75 kg patient per liter of infusion. Some authors recommend limiting initial energy uptake to 10 kcal/kg/day in patients at risk of RFS [4,35]. Nonetheless, our recommendation is not to decrease the energy intake but to supplement the missing electrolytes in older patients requiring rehydration therapy. According to our findings, the patients at risk developed only to mild or moderate plasma deficiencies due to the early supplementation of electrolytes, in concordance with Reber et al. [29]. Furthermore, the in-hospital mortality rates for the P group (16.7%) and G group (18.8%) were comparable to the 22.5% reported by Lubart et al. [31]. This suggests that complications from refeeding syndrome (RFS) were effectively managed via careful laboratory monitoring, an approach we advocate for within the first 72 h of admission.

Elevated parenteral supplementation of intracellular ions (phosphates, potassium, magnesium) corresponded with reduced urinary excretion, suggesting cellular uptake and the initiation of anabolic processes [11]. Consistent with this, Winter et al. observed enhanced whole-body protein synthesis during refeeding in patients with illness-induced starvation [26]. These findings support the safety of higher initial caloric intakes.

There are some limitations of our study. First, our patients were medical inpatients at standard medical wards. We did not include patients at intensive care units. This may explain the discrepancy in the results of our study compared to those of Doig [36] and Olthof [37], where restricted caloric intake in critically ill patients with refeeding syndrome was associated with better survival than normal caloric intake. Second, we did not test for patients´ malabsorption, as described in other works [38,39].

In our study, the glucose intake during rehydration led to anabolic shifts of minerals typical of refeeding syndrome. However, despite the recognized role of glucose in the pentose phosphate pathway and its potential anabolic benefits, energy provision alone was not sufficient to improve outcomes [40], likely due to prevalent insulin and anabolic resistance in older adults during acute illnesses [41,42]. Possibly, glucose should be supplemented together with an increased protein intake and exercise to enhance anabolism [14,41,43,44,45]. Further study is necessary to elucidate our questions.

## 5. Conclusions

In conclusion, early glucose supplementation showed no detrimental effects and is deemed safe and potentially beneficial to initiate intracellular anabolism. This challenges the conventional approach of caloric restriction due to RFS fears and underscores the importance of monitoring intracellular ion levels closely within the first 72 h to guide ion supplementation strategies effectively, rather than glucose discontinuation and caloric restriction.

## Figures and Tables

**Table 1:** Composition of administered intravenous crystalloid solutions.

Constituent ^1^	PlasmaLyte (P)	Added Glucose (G)
Na^+^	140	140.1
K^+^	5	5.1
Ca^2+^	0.8	0.6
Mg^2+^	1.5	1.1
Cl^−^	98	110.8
P^5−^	-	-
Gluconate	23	16.9
Acetate	27	19.8
Glucose (g L^−1^)	0	100.9
Osmolality (mOsm L^−1^)	295	832

^1^ Unless stated otherwise, the units are mmol L^−1^.

**Table 2:** Cohort characteristics.

Parameter	PlasmaLyte (P)	Glucose (G)
Number of patients (n)	18	16
Age	85.0 (3.5) ^1^	85.0 (6.8)
Men/women	9/9	3/9
Body mass index	26.0 (5.2)	24.5 (4.3)
Clinical signs of dehydration at admission	3.5 (1.3)	3.5 (1.8)
Number of acute diagnoses at admission	5.0 (3.5)	5.0 (4.0)
Length of hospital stay	11.5 (5.8)	9.0 (6.5)
In-hospital mortality	3 (16.7)	3 (18.8)
Barthel’s index (ADL)	10.5 (50.5)	22.5 (62.5)
Norton’s score of pressure ulcer risk	18.5 (18.3)	20.0 (26.0)
Baseline glycaemia (mmol L^−1^)	7.6 (2.4)	7.2 (3.5)
Baseline osmolality (mOsm L^−1^)	284.0 (15.0)	286.5 (22.0)
Baseline natrium (mmol L^−1^)	138 (5.0)	137 (7.8)
Baseline potassium (mmol L^−1^)	4.0 (0.7)	3.9 (0.8)
Baseline magnesium (mmol L^−1^)	0.74 (0.14)	0.86 (0.15)
Baseline phosphates (mmol L^−1^)	0.94 (0.26)	0.99 (0.27)
Baseline urea (mmol L^−1^)	7.0 (6.6)	7.8 (3.7)
Baseline creatinine (µmol L^−1^)	87.0 (26.0)	102.5 (70.0)
Baseline CRP (mg L^−1^)	78.1 (132.8)	58.3 (99.4)
Baseline leukocyte count (×10^9^ L^−1^)	10.6 (6.5)	10.7 (7.0)

^1^ Unless stated otherwise, the values are expressed as median (interquartile range). Frequency data are provided as absolute number (percentage). There was no significant difference in any of the parameters.

## Data Availability

Our study did not use publicly archived datasets. The original contributions presented in the study are included in the article and its Supplementary Materials, further inquiries can be directed to the corresponding authors.

## Supplementary Materials

The following supporting information can be downloaded at: https://www.mdpi.com/article/10.3390/nu16111607/s1. Figure S1: calculation of slopes for individual patients. Figure S2: 72 h trends of electrolytes. Figure S3: weekly trends of electrolytes. Figure S4: comparison of electrolyte substitution. Figure S5: weekly trends of markers of inflammation.
