# Supplementary figures and images for: Optimizing Recovery in Elderly Patients: Anabolic Benefits of Glucose Supplementation during the Rehydration Period
Source: Nutrients. 2024 May 24;16(11):1607. doi: 10.3390/nu16111607 (PMC11173922; doi:10.3390/nu16111607)

# Patient ID #02 G

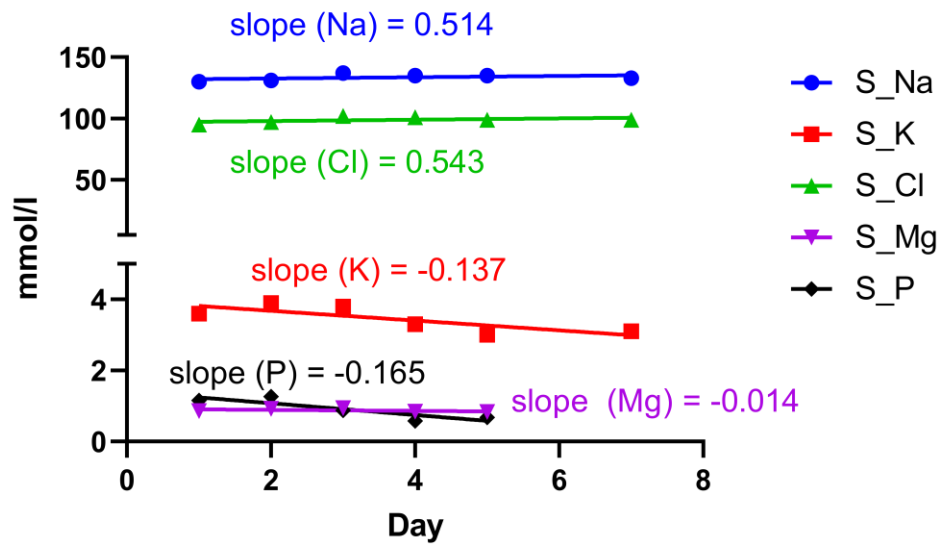

## Supplementary Figure 2 - 72-h trends

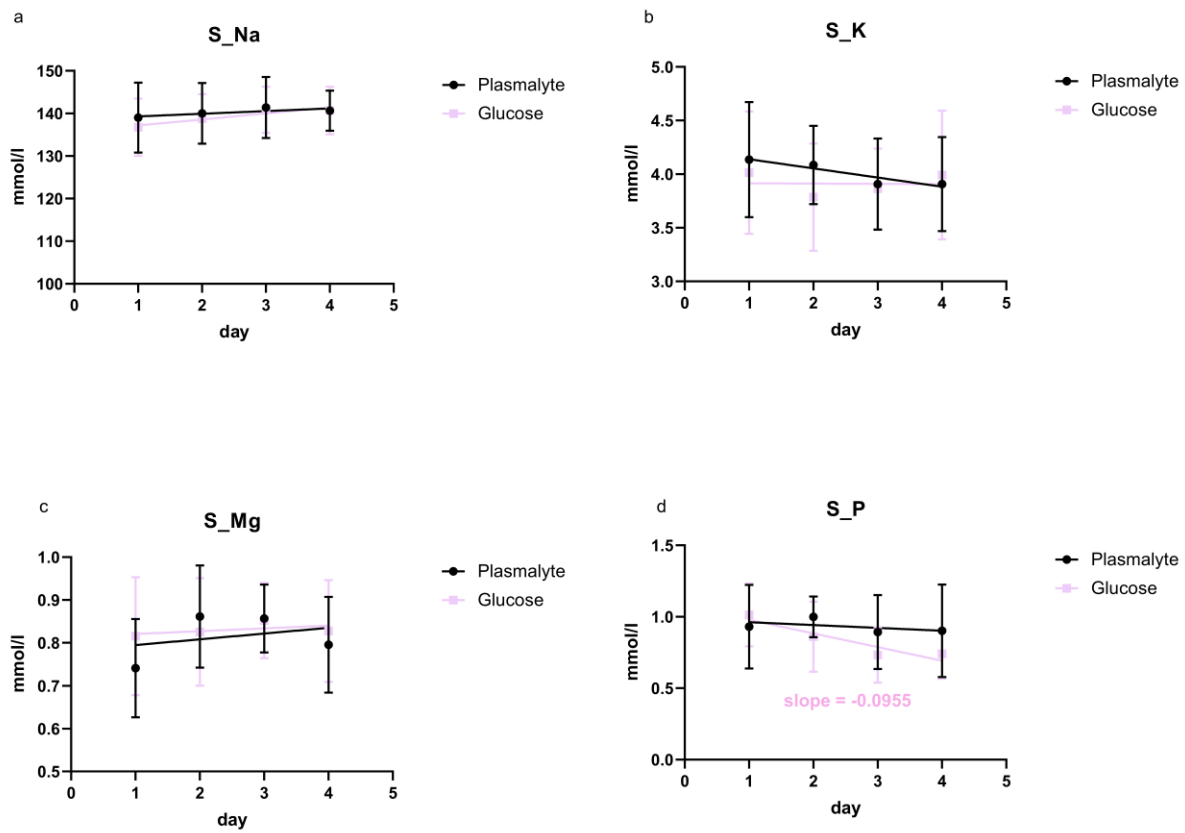

## Supplementary Figure 3 - weekly trends

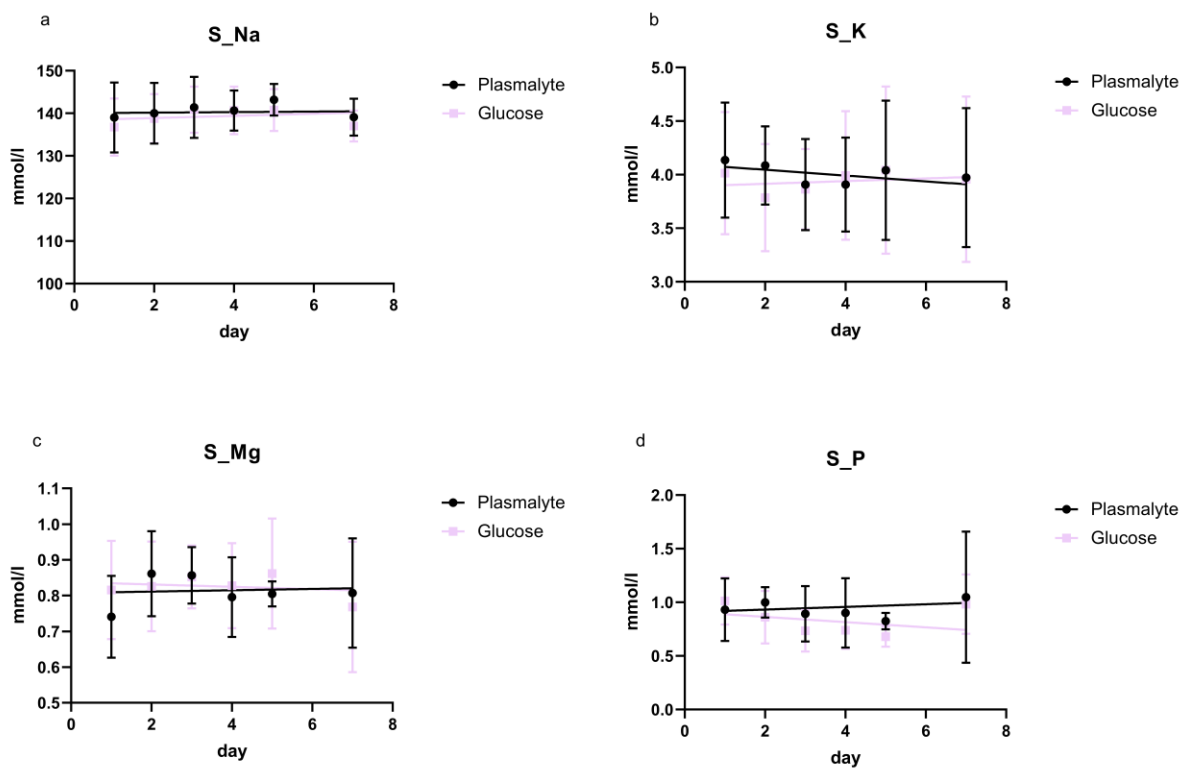

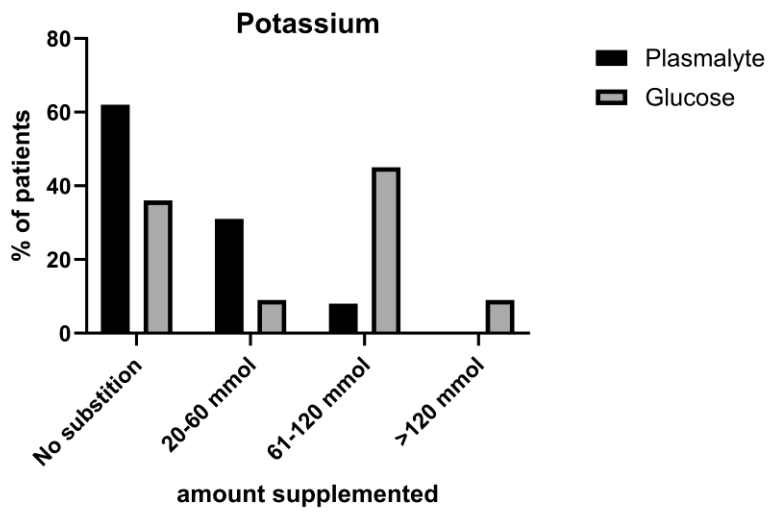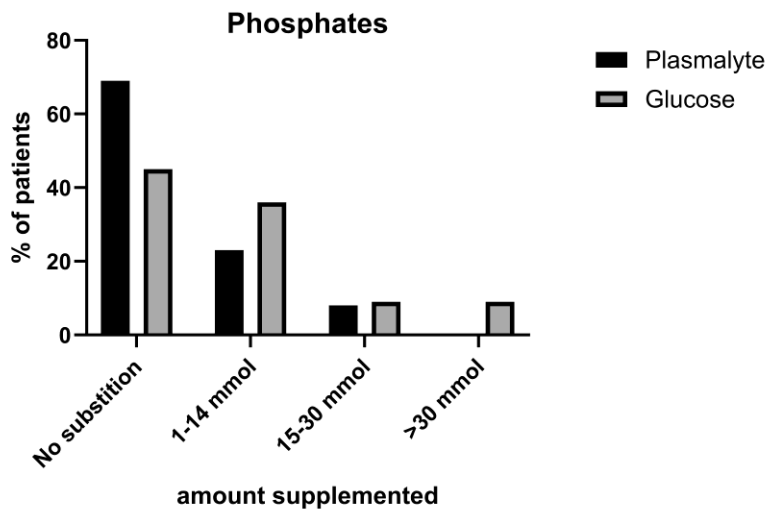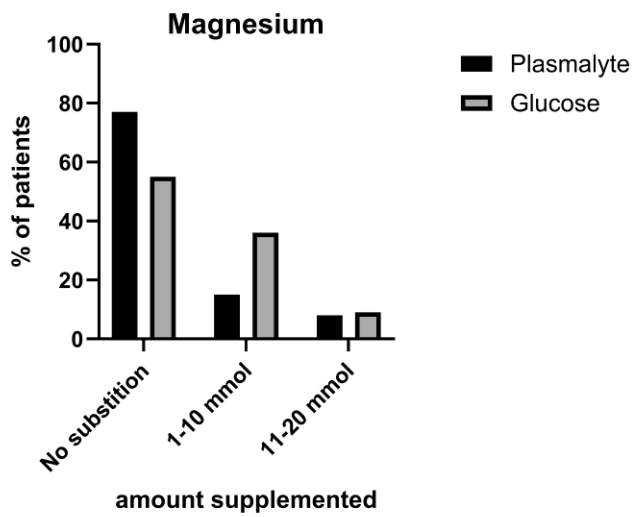

Supplement: Supplementary file 1 [file nutrients-16-01607-s001.zip › nutrients-3006938-supplementary.pdf]
